# A Dual Role for the Dorsolateral Prefrontal Cortex (DLPFC) in Auditory Deviance Detection

**DOI:** 10.3390/brainsci14100994

**Published:** 2024-09-29

**Authors:** Manon E. Jaquerod, Ramisha S. Knight, Alessandra Lintas, Alessandro E. P. Villa

**Affiliations:** 1 NeuroHeuristic Research Group, University of Lausanne, Quartier UNIL-Chamberonne, 1015 Lausanne, Switzerlandalessandra.lintas@unil.ch (A.L.); 2 Beckman Institute, University of Illinois at Urbana-Champaign, 405 N Mathews Ave., Urbana, IL 61801, USA; 3 Aptima, Inc., 2555 University Blvd, Fairborn, OH 45324, USA; 4 LABEX, HEC Lausanne, University of Lausanne, Quartier UNIL-Chamberonne, 1015 Lausanne, Switzerland

**Keywords:** optical imaging, frequency-domain fNIRS, oddball paradigm, mismatch negativity, BA 46, BA 8, P300

## Abstract

Background: In the oddball paradigm, the dorsolateral prefrontal cortex (DLPFC) is often associated with active cognitive responses, such as maintaining information in working memory or adapting response strategies. While some evidence points to the DLPFC’s role in passive auditory deviance perception, a detailed understanding of the spatiotemporal neurodynamics involved remains unclear. Methods: In this study, event-related optical signals (EROS) and event-related potentials (ERPs) were simultaneously recorded for the first time over the prefrontal cortex using a 64-channel electroencephalography (EEG) system, during passive auditory deviance perception in 12 right-handed young adults (7 women and 5 men). In this oddball paradigm, deviant stimuli (a 1500 Hz pure tone) elicited a negative shift in the N1 ERP component, related to mismatch negativity (MMN), and a significant positive deflection associated with the P300, compared to standard stimuli (a 1000 Hz tone). Results: We hypothesize that the DLPFC not only participates in active tasks but also plays a critical role in processing deviant stimuli in passive conditions, shifting from pre-attentive to attentive processing. We detected enhanced neural activity in the left middle frontal gyrus (MFG), at the same timing of the MMN component, followed by later activation at the timing of the P3a ERP component in the right MFG. Conclusions: Understanding these dynamics will provide deeper insights into the DLPFC’s role in evaluating the novelty or unexpectedness of the deviant stimulus, updating its cognitive value, and adjusting future predictions accordingly. However, the small number of subjects could limit the generalizability of the observations, in particular with respect to the effect of handedness, and additional studies with larger and more diverse samples are necessary to validate our conclusions.

## 1. Introduction

The orientation of selective attention towards unusual events is a fundamental ability that helps living organisms ensure their survival [1,2]. A classical and well-studied experimental protocol for examining brain interactions with the perceptual environment is the auditory oddball paradigm. In this paradigm, a standard (frequent) stimulus is repeated at fixed intervals and occasionally replaced, with low probability, by a deviant stimulus [3,4]. The conscious perception of sound involves activating a complex network of brain regions, which convey auditory information from low-order sensory processing areas to higher-order processing regions in the frontal lobes [5,6,7]. The presentation of standard sounds (high-probability stimuli) necessitates detecting regularities in the sequence of auditory stimuli, whereas the presentation of a deviant sound (low-probability stimulus) requires detecting a prediction error—i.e., a mismatch between expected and perceived auditory stimuli.

Several event-related potential (ERP) components, including N1, P2, N2, and P3, are time-locked to stimulus onset and exhibit varying latencies and amplitudes between deviant and standard stimuli [4,8,9,10,11,12]. The N1 response, occurring 100–170 ms after sound onset, is linked to acoustic feature and change detection and is enhanced during selective attention tasks [10,13,14,15]. The N1–P2 complex characterizes the auditory response to the standard stimulus. The P2 response, elicited at 190–240 ms post-sound onset, reflects further stimulus evaluation and classification and is typically enhanced following prolonged training [12,16,17,18]. A mismatch between an expected standard stimulus and an incoming deviant stimulus triggers a negative shift in the ERP around 200–250 ms (N2) from stimulus onset. Subtracting ERP responses to standard and deviant stimuli highlights this wave component, known as mismatch negativity (MMN), which is recorded at frontocentral and central sites [10,19,20]. Studies in awakened, freely moving animals have demonstrated the presence of MMN-like responses in the auditory cortex of cats [21,22] and rats [23] showing that non-human animals are capable of detecting auditory deviations associated with a cognitive value. The work by Nelken and Ulanovsky [24] extended these findings, showing that auditory deviance detection in animals shares similarities with human auditory processing, offering a valuable comparative perspective on MMN physiology.

At least two sources have been proposed as generators of MMN: a bilateral supratemporal source responsible for pre-perceptual change detection and a frontal source associated with an involuntary attention switch toward the deviant stimulus [25,26]. In the passive version of the oddball paradigm, participants listen to the sequence of auditory stimuli without responding. In contrast, in the active version, participants are required to respond when deviant stimuli are detected, which elicits a P3 (or P300) component with a larger amplitude than in passive conditions. A shift in selective attention, often triggered by the detection of deviant stimuli, is commonly observed when comparing active and passive versions of the paradigm [27,28,29]. This shift is associated with a frontocentral N2–P3a complex, followed by a temporoparietal P3b wave that occurs later in active conditions than in passive conditions [29,30,31]. Auditory information is processed along two parallel pathways in the cerebral cortex: the anterior-temporal-to-inferior-frontal pathway (the “what” pathway) and the parietal-to-lateral-prefrontal pathway (the “where” pathway) [32,33,34]. While these pathways are crucial for general auditory perception, the dorsolateral prefrontal cortex (DLPFC), part of the “where” pathway including Brodmann’s areas (BA) 8, 9, and 46, plays a key role in cognitive tasks involving deviance detection and working memory [35]. Additionally, DLPFC activation, coupled with MFN following the passive detection of deviant stimuli, has been suggested to be related to error prediction between the expected and actual stimulus [36]. The relatively high spatial resolution (but limited temporal resolution) of functional magnetic resonance imaging (fMRI) has confirmed sustained activity in extensive networks of brain areas during the auditory oddball paradigm [37]. An fMRI study demonstrated that dorsal prefrontal activity increases with lower target frequencies, while ventral prefrontal cortex activity increases with higher target frequencies [38]. However, blood-oxygen-level-dependent (BOLD) fMRI signals may not be strong enough to detect the activation (or inhibition) of spatially confined neuronal populations that generate significant ERP waves.

Functional frequency-domain near-infrared spectroscopy (FD-fNIRS) is a non-invasive optical brain imaging technique characterized by its limited penetration depth (a maximum of 3–5 cm from the skull), with temporal resolution on the order of milliseconds and spatial resolution with voxel diameters of a few millimeters [39]. Event-related optical signals (EROS), similar to event-related potentials (ERPs), are derived by averaging fast optical measurements triggered by stimulus onset. Neurovascular signals are generated by changes in the properties of infrared laser light photons interacting with oxygen-bound hemoglobin [40,41,42,43]. In addition, EROS detects a neurogenic signal, which is linked to modifications in photon scattering caused by neural activity, such as the firing of neurons [44,45,46,47]. The concurrent use of EROS with other imaging techniques has become increasingly popular in recent years [48,49,50,51,52,53,54]. In various oddball paradigms, EROS changes have been reported to co-occur with mismatch negativity (MMN) in the left [54,55] and right supratemporal gyri (STGs) [48,49,56,57], as well as in the right inferior frontal gyrus (IFG) [48,57]. In active oddball protocols, task-dependent effects have been observed in both EROS and the P3 component of ERPs, notably in the superior frontal gyrus (SFG) [55] and the middle frontal gyrus (MFG) [58].

Previous studies have primarily explored the DLPFC’s involvement in active cognitive responses, such as maintaining information and adapting strategies [58,59,60], but less is understood about its role in passive auditory perception. Our study aims to address this gap by investigating the DLPFC’s activity during passive auditory deviance detection using a combination of event-related potentials (ERPs) and event-related optical signals (EROS), using for the first time, to the best of our knowledge, a 64-channel electroencephalography (EEG) system with FD-fNIRS. We hypothesize that the DLPFC not only participates in active tasks but also plays a critical role in processing deviant stimuli in passive conditions, shifting from pre-attentive to attentive processing. We detected enhanced neural activity in the left MFG (BA 46), at the same timing of the MMN component, followed by later activation in the right MFG (BA 8), at the timing of the P3a ERP component. Understanding these dynamics will provide deeper insights into the DLPFC’s dual role in cognitive and sensory processing.

## 2. Methods

### 2.1. Participants

A total of thirteen right-handed healthy young adult volunteers were initially recruited for this experiment, with handedness laterality being a critical selection criterion to avoid known effects on auditory perception [61]. This sample included 6 participants from a preliminary study used to adjust the concurrent recording settings of EROS and ERPs [62]. Before participation, all subjects were informed about the procedures and provided signed informed consent in accordance with the Declaration of Helsinki [63] and the ethical and data security guidelines of the University of Lausanne (ethics approval by Swiss Association of Research Ethics Committees CCER-20190095). One participant was excluded post-recruitment due to previously undisclosed left-handedness. Consequently, the final sample for this study comprised twelve participants (7 women and 5 men) with an average age of 26.6 and a standard error of the mean (SEM) equal to 1.2 years.

### 2.2. Behavioral Task

The task followed the passive auditory oddball paradigm and consisted of 12 blocks, each containing 120 trials. In each block, a randomized sequence of digitally generated pure tones was presented, with each tone lasting 500 ms. The tones were classified as standard (*frequent*, 1000 Hz, with an occurrence probability of *p* = 92%) and deviant (*rare*, 1500 Hz, *p* = 8%). The interval between the onset of two successive stimuli was set to 1600 ms. Auditory stimuli were delivered through small loudspeakers placed immediately behind the participants. The sound level was set to approximately 60 ± 2 dB SPL, measured using a digital sound level meter (TES1350, TES Electrical Electronic Corp., Taipei, Taiwan) positioned at the participants’ ear level. Headphones could not be used for the presentation of sounds due to interference with the helmet used for EEG/TD-fNIRS recordings (see Figure 1 in Jaquerod et al. [62]). Participants were instructed to direct their attention towards a white cross on a black background displayed at the center of a computer monitor, positioned 65 cm from their eyes. Experiments were conducted in a quiet test room [64] and in complete darkness, with all lights turned off to minimize noise in the optical data acquisition.

### 2.3. EEG Recording and ERP Processing

Continuous EEG was recorded using 64 scalp Ag/AgCl active electrodes from an ActiveTwo MARK II Biosemi EEG System (BioSemi B.V., Amsterdam, The Netherlands) with a sampling rate of 1024 Hz, referenced to the linked mastoids. Impedance for all recordings was maintained below 20 kΩ. Two head caps of different sizes (NeuroSpec Quick Cap, NeuroSpec AG, 6370 Stans, Switzerland), following the 10/20 layout, were used to accommodate small (<56 cm) and medium–large (≥56 cm) head circumferences. EEG signals were preprocessed and analyzed using the EEGLAB [65] (version 14.1.2b) and ERPLAB [66] (version v8.30) toolboxes in Matlab© version 2021b (The MathWorks, Inc., Natick, MA, USA). Raw signals were bandpass-filtered between 0.1 Hz and 40 Hz with a −36 dB/octave roll-off.

The EEG data were segmented into epochs marked by the onset of auditory stimuli. Epochs with visible large movement artifacts were excluded from further analysis. The EEG signal was decomposed using an Infomax Independent Component Analysis (ICA) for correction of eye blinks, eye saccades, muscle activity, and other interference artifacts identifiable in the ICA components. Epochs containing visible artifacts post-ICA preprocessing were also discarded. For both standard and deviant stimuli conditions, epochs were averaged, with a 160 ms interval prior to stimulus onset used for baseline correction. Local peaks within a selected time window of interest were determined with the ERP Measurement Tool of ERPLAB [66] and “local peak latency” and “local peak amplitude” were measured accordingly. The N1 ERP component local peak was identified at the lowest voltage between 100 and 200 ms post-stimulus onset, N2 as the locally lowest voltage in the 200–270 ms interval, and the P3a and P3b local peaks were identified at the highest voltage in the 270–340 ms and 340–420 ms intervals, respectively. Average ERPs for each participant were normalized separately for each electrode based on individual peak-to-peak amplitude to account for between-subject variability in electrode impedance.

We defined 3 sets of frontal electrodes (left: F3, F5, FC1, FC3; midline: F1, Fz, F2, FCz; right: F4, F6, FC2, FC4), 3 sets of central electrodes (left: C3, C5, CP1, CP3; midline: C1, Cz, C2, CPz; right: C4, C6, CP2, CP4) and 3 sets of posterior electrodes (left: CP5, P3, P5; midline: P1, P2, Pz; right: CP6, P4, P6). Poor ERP signals from electrodes included in the sets were identified using the average standardized measurement error (SME) [67]. Electrodes with an average SME > 3 microvolts (μV) were excluded.

### 2.4. Optical Recording and EROS Processing

**Figure 1:**
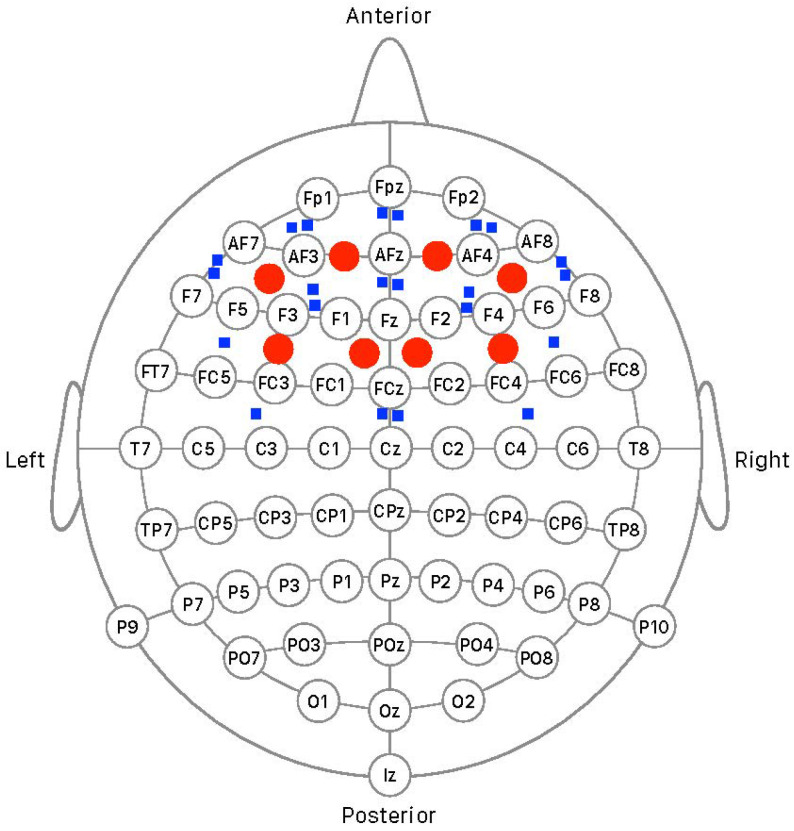
Schematic representation of the co-localization of the 8 light detectors (red circles) and 22 light sources (blue squares) over prefrontal and premotor areas of the cerebral cortex and the 64-channel EEG setup according to the International 10/20 system.

Optical recording was performed using a frequency-domain NIRS system, the ISS Imagent (Champaign, IL, USA), equipped with 8 light detectors (photomultiplier tubes) and 22 frequency-modulated laser light sources (830 nm wavelength modulated at 110 MHz). The optical probes were co-located with the EEG setup, as depicted in Figure 1. Custom-built helmets were designed to hold the fiber-optic bundles connected to the laser diodes emitting the light sources and the fiber-optic bundles connected to the detectors in place. Two helmet sizes were constructed—one small and one medium–large—to integrate the optical equipment with the small and medium–large EEG head caps, which were modified to allow direct contact of the optical probes with the scalp. At the conclusion of the experiment, the precise locations of each optical probe and EEG recording site were digitized using a FASTRAK 3Space digitizer (Polhemus Inc., Colchester, VT, USA). The source-to-detector distance is a critical parameter for determining the validity of optical recordings. Distances that are too short may not be compatible with an intracranial origin of the signal, and distances that are too long may not capture sufficient photons for a valid measurement [68]. In this experiment, we only considered source-to-detector distances in the range of 20–55 mm.

Detector amplifiers were modulated at a frequency of 110.005 MHz [69]. Consequently, a heterodyning frequency (or cross-correlation frequency) was generated, equal to the difference between the frequency modulations of the sources and detectors, i.e., 5000 Hz. This implies an oscillation period of 0.2 ms. The photomultiplier output current underwent a Fast Fourier Transform (FFT) over four oscillations (0.2 ms × 4=0.8 ms). To avoid cross-talk between sources during data acquisition, one additional oscillation was skipped, yielding a period of 1.0 ms for the acquisition from each source. The light sources were time-multiplexed in cycles of eight per sampling point, resulting in an effective time resolution of 8 ms (equivalent to an effective sampling rate of 125 Hz). For each data point, we measured the DC (average) intensity, AC (amplitude) intensity, and relative phase delay. The optical recordings were processed following a workflow similar to that used by Low et al. [58]. Initially, this process involved removing drifts in phase delay measurements of the cross-correlation signal to adjust the mean phase delay measurements to zero. Subsequently, pulse artifacts were removed, and the data were filtered using a sixth-order Butterworth band-pass filter with settings from 0.5 to 10 Hz. Event-locked epochs, starting 160 ms before and extending up to 640 ms after stimulus onset (i.e., an overall interval of 800 ms), were averaged across conditions and checked for signal values within a valid signal-to-noise range. Following previous analyses performed with the same type of equipment [68,69], we discarded signals from channels (each pair of light source and detector is a channel) with a standard deviation in phase delay greater than 160 ps.

Individual average phase delay measurements in the voxel space (raw data) were extracted and used to compute the EROS Grand Average (across all participants for a specific condition). Additionally, a standardization procedure was applied to account for optical signal variability between participants. For each participant, each voxel curve was processed by dividing the value at each time point by the standard deviation computed across all time points. EROS data were then standardized using a peak-to-peak amplitude-based procedure similar to that used for averaged ERPs, but the lowest and highest amplitudes corresponding to the local peak values were determined in the interval from 0 to 640 ms after stimulus onset.

### 2.5. Brain Mapping and Regions of Interest

Highly accurate individual 3D digitization files of skull sites, obtained with the Polhemus FASTRAK 3Space digitizer, were co-registered with three sizes of structural MRI using the Optimized Co-registration Package software, Version 1.0 [70]. For each participant, we selected the co-registration that minimized the fiducial alignment error for further analysis. The optical signals, originally in channel space, were projected onto an MRI brain template. This projection followed a physical homogenous model of photon propagation through tissue, using Opt3D software, version 10 [71], available at the NeuroImaging Tools & Resources Collaboratory (https://www.nitrc.org/, accessed on 27 September 2024). EROS data were spatially filtered with an 8 mm Gaussian kernel. The regions of interest (ROIs) in this study were limited to six areas, namely, BA8, BA9, and BA46 in the DLPFC of each hemisphere. The projected voxel space is centered on the anterior commissure to align with the Talairach atlas *x*–*y*–*z* coordinates and to facilitate ROI labeling using the Talairach Daemon database label server, distinguishing between the left and right hemispheres [72]. A file in the NIfTI format was created for each of the six ROIs and uploaded to Opt3D for the purpose of correcting for multiple comparisons within each ROI.

### 2.6. Statistical Analysis

R 4.3.2 statistical software was used in all the analyses [73]. In general, the grouped values are reported as (median, average ± SEM). Factorial analyses are performed with Type-III ANOVA and the generalized eta squared ($\eta_{G}^{2}$) is used to test the effect size. The magnitude of effect sizes is considered as negligible if $\eta_{G}^{2}<0.01$, small for $0.01\leq \eta_{G}^{2}<0.06$, medium for $0.06\leq \eta_{G}^{2}<0.14$, and large if $0.14\leq \eta_{G}^{2}$. Group-level *t*-statistics of a differential EROS response in phase delay between conditions were computed and converted into Z-scores. Power analysis was run using the R package pwr [74].

## 3. Results

### 3.1. Analysis of the Local Peaks in the ERP Components

**Figure 2:**
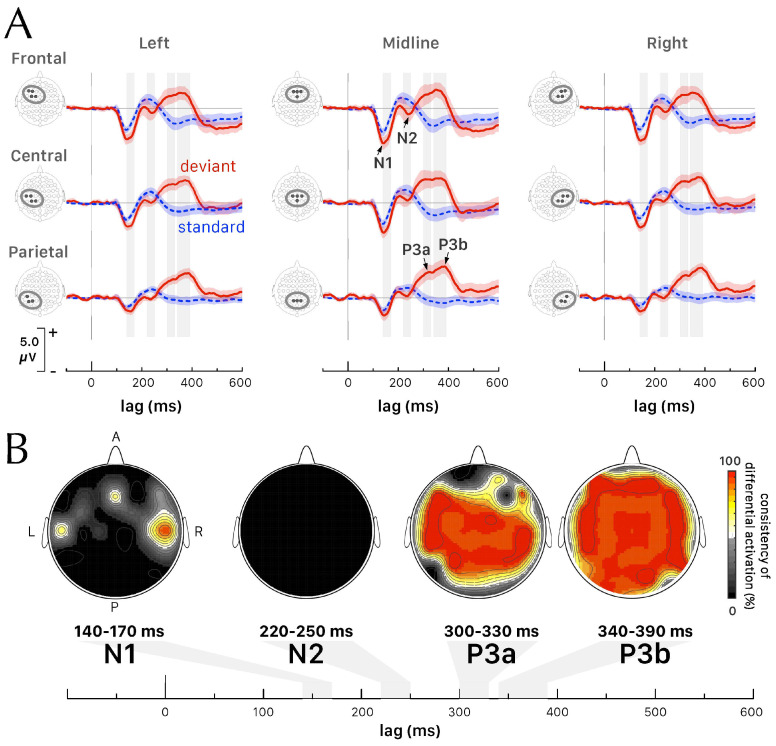
(**A**) Grand–average waveforms of the ERPs evoked by standard (dashed blue) and deviant (red) tones (mean ± 2 × SEM) at locations corresponding to 9 sets of electrodes along the antero-posterior and mesio-lateral axis. Four ERP components (N1, N2, P3a, and P3b) were identified. (**B**) Topographic maps of the consistency of differential activations (contrast analysis between deviant and standard tone conditions) for N1, N2, P3a, and P3b ERP components. The contour lines connect the points with the same value of consistency of differential activation.

We applied a very stringent artifact rejection criteria to the EEG recordings. The mean (median) number of valid trials was equal to ${\hat{n}}_{\mathrm{standard}}=$ 898 (904) out of 1325 standard-tone trials and ${\hat{n}}_{\mathrm{standard}}=$ 95 (92) out of 115 deviant-tone trials. Both conditions evoked a negative peak in the ERP corresponding to the N1 component, identified as the lowest voltage within the 100–200 ms window (Figure 2A). Three other ERP components, corresponding to the N2 (Mismatch Negativity, MMN) and P3a and P3b waves, were evoked only after the deviant tone (Figure 2A).

The grand average measurements (across participants and across sets of electrodes) of the N1 local peak ERP component yielded *latency*($N1_{standard})\;=\;143.4\;$± 0.8 ms vs. *latency*($N1_{deviant})\;=\;146.9\;$± 0.9 ms and *amplitude*($N1_{standard})\;=\;-3.25\;$± 0.16 μV vs. *amplitude*($N1_{deviant}\;=\;-5.23\;$± 0.24 μV. We observed that N1 local peak latencies varied between 132 and 166 ms after stimulus onset in both conditions and that the amplitude tended to be maximal over frontocentral electrode sites. We computed an analysis of variance (ANOVA) for the latency and amplitude of the N1 local peak with a 2×3×3 factorial design, i.e., *tone* (’deviant’, ’standard) × *anteriority* (’frontal’, ’central’, and ’parietal’) × *laterality* (’left’, ’midline’, ’right’). We observed a small main effect of the tone on the N1 peak latency (F(1, 198)=11.86, p= 0.001, $\eta_{G}^{2}=0.054$, post hoc power=0.919) with no interaction effects of any kind between the factors. For the N1 peak amplitude, we observed no interaction effects of any kind between the factors and meaningful independent main effects for the tone and anteriority (F(1, 198)=42.21, p< 0.001, $\eta_{G}^{2}=0.129$, power=1 and F(2, 198)=37.76, p< 0.001, $\eta_{G}^{2}=0.231$, power=1, respectively). In particular, the main effect of anteriority explained 23.1% of the variance in amplitude of N1 peak, indicating a strong effect size. A small main effect on N1 amplitude might be observed also for the laterality (F(2, 198)=3.60, p= 0.029, $\eta_{G}^{2}=0.035$, power=0.667).

A 3×3 factorial design ANOVA, i.e., *anteriority* (’frontal’, ’central’, and ’parietal’) × *laterality* (’left’, ’midline’, ’right’), was used to test the latency and amplitude of the local peaks for the three components observed only during deviant tone trials. No effect of laterality and no interaction effect were observed for any of the ERP components evoked only by deviant tones. For N2, a small main effect of anteriority on the peak amplitude could be observed (F(2, 82)=3.61, p= 0.031, $\eta_{G}^{2}=0.080$, power=0.664). Conversely, we observed a medium main effect of anteriority on the latency of P3a (F(2, 94)=5.45, p= 0.006, $\eta_{G}^{2}=0.102$, power=0.841). For P3b, the main effect of anteriority was significant on both latency and amplitude variables (F(2, 99)=6.70, p= 0.002, $\eta_{G}^{2}=0.115$, power=0.905 and F(2, 99)=8.94, p< 0.001, $\eta_{G}^{2}=0.151$, power=0.972, respectively).

### 3.2. Differential Activations between Conditions in the ERPs

We conducted a contrast analysis of the ERPs between deviant and standard tone conditions. This involved comparing the amplitude values of the grand–average ERPs (averaged across participants) recorded at each site within the 64-channel EEG setup. We applied the Bonferroni multiple testing correction method to ensure that the adjusted *p*-value ($p_{adjusted}$) was less than 0.05. For each electrode site and each time point within a selected time interval, we determined the differential activation. This differential activation was defined as 1 if there was a significant difference in ERP amplitudes between deviant and standard tones, and 0 otherwise. We then computed the consistency of differential activation for each electrode site over the selected time interval. This was accomplished by averaging the values of the differential activation across all time points within the interval. A consistency of differential activation equal to 100% indicates that, at every time point within the selected interval, the ERP amplitudes for standard and deviant tones were significantly different, with $p_{adjusted}<0.05$. A value of consistency of differential activation equal to 50% means a differential activation that is significant during half of the time points over the selected time window. We have computed topographic maps by integrating the values of consistency of differential activation for all electrode sites over time intervals 140–170 ms for N1, 220–250 ms for N2, 300–330 ms for P3a, and 340–390 ms for P3b (Figure 2B).

For N1, Figure 2B shows significant differential activations around electrode sites C6, and to a lesser extent at C5 and Fz. The central electrode sets display a greater difference in the latency of N1 evoked by the deviant tone compared to the standard tone, particularly for the lateral sets of electrodes compared to the midline (t(11)=2.831, p= 0.016 with a Cohen’s *d* effect size of 0.82, indicating a large effect). The differential activation shown on Figure 2B at site Fz alone for N1 was very localized and no difference was statistically significant when the test was performed with the whole frontocentral set of electrodes, which included F1, F2, and FCz in addition to Fz. No significant differential activation was observed between the conditions during the N2 ERP component time window. This is attributed to the similar variance in the mismatch negativity amplitude in the deviant tone condition and the ERP evoked by the frequent tone in the 220–250 ms interval. Conversely, the deviant tone’s large P300 component generated a widespread pattern of significant differential activations across the scalp, with the maximum activation occurring at P3b between 340 and 390 ms after tone onset.

### 3.3. Differential Activations between Conditions in the Fast Optical Signals

**Figure 3:**
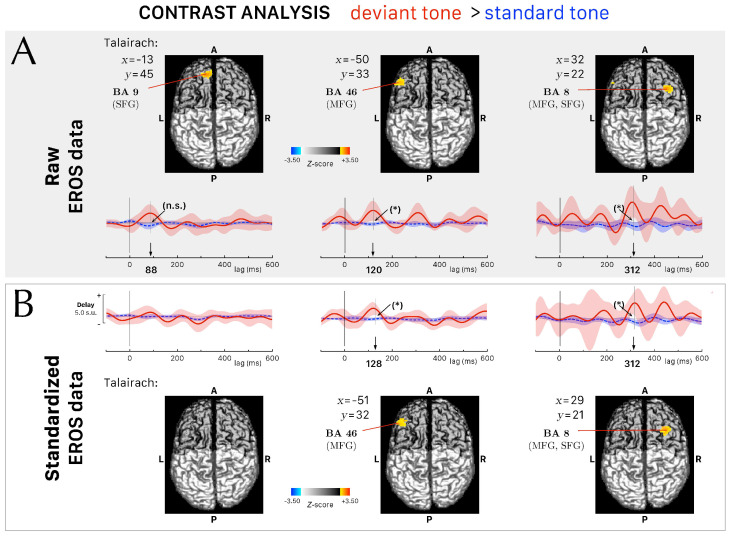
Differential activations in the event–related optical signals (EROS) following a contrast analysis (*deviant tone* > *standard tone*, Z-score >2.575, n > 10. (**A**) Raw EROS data analysis. The upper panels show the axial projection of the Z-score surface maps (computed across subjects) on a template MRI for the contrast analysis at 88, 120, and 320 ms after stimulus onset. The Talairach coordinates *x* and *y* of the voxels with the greatest differential activation are indicated with the corresponding Brodmann area (BA) and nearest cortical gyri. The corresponding Talairach *z* coordinate is on the cortical surface. The lower panels show the corresponding EROS grand–average curves (mean ± 2 × SEM), from 100 ms before stimulus onset to 600 ms after stimulus onset. of the peak voxel and its direct neighboring voxels during the deviant (red) and the standard (dashed blue) tones conditions. An arrow indicates the timing of the greatest differential activation with a sign (n.s.) not significant and (*) p<0.05, for the significance level of the differential activation at the peak latency with multiple comparison correction within the associated ROI. (**B**) Standardized EROS data analysis. The upper panels show the axial projection of the new Z-score surface maps (computed across subjects) on a template MRI recomputed following the standardization procedure described in the Methods Section 2.4, for the contrast analysis at 88, 128, and 320 ms after stimulus onset. At 88 ms, no voxel of the differential activation reached the threshold level Z-score >2.575, n > 10. At 128 and 320 ms after stimulus onset, the Z-score of the differential activations was above the threshold level and remained significant (p<0.05) even after multiple comparison correction within the associated ROI.

For each participant, we recorded fast optical signals with a standard deviation in phase delay lower than 160 ps from 32 channels on average (*min* = 21, *max* = 50). The contrast analysis (deviant > standard) revealed three intervals of interest (each one lasting 24–32 ms) with a significant group-level difference in phase delay responses between conditions (Table 1, Z-score >2.575, n > 10). The greatest difference in the first interval peaked at 88 ms post-stimulus onset in the left SFG (BA 9). Figure 3A shows the associated EROS curve with a a positive deflection—i.e., an increase in the phase delay–in the deviant tone condition (red curve) peaking at 88 ms, with a Z-score =2.95. However, after computing the critical Z-scores ($Z_{crit}=3.11$) for a 5% significance level with multiple comparison correction within the associated ROI (Table 1), the differential activation at 88 ms is no more significant.

The second interval of interest comprised between 112 and 136 ms after stimulus onset. The greatest difference peaked at 120 ms post-stimulus onset in the left MFG (BA 46) (Figure 3A). This differential activation is significant after the correction for multiple comparison ($Z=2.76>Z_{crit}=2.67$). It is interesting to note the robustness of this peak in the differential activation, which remains significant after the correction for multiple comparison also in the analysis of standardized phase delays (Figure 3B). The last interval of interest shows a significant differential activation peaking at 312 ms post-stimulus onset in the left hemisphere, in an area near the borders of MFG and SFG (BA 46) after the correction for multiple comparison (Table 1) in the analyses of both raw (Figure 3A) and standardized phase delays (Figure 3B).

## 4. Discussion

The most significant theoretical contribution of this study lies in the integration of ERP and EROS to provide a more comprehensive understanding of brain dynamics during auditory deviance detection. Our study builds on recent studies [75,76,77], that demonstrated the value of combining multimodal imaging techniques. This dual measurement approach allows for the simultaneous assessment of both the timing (with ERP) and localization (with EROS) of neural responses, which is not possible with either technique alone. Specifically, the observed early activation in the left DLPFC (BA 46), corresponding to MMN, suggests that this region is involved in pre-attentive, predictive coding processes. In contrast, the later activation in the right DLPFC (BA 8), associated with the P300 component, points to this region’s involvement in evaluating and updating the cognitive significance of the deviant stimulus. These findings align with and extend predictive coding theories, which suggest that the brain continuously updates its predictions based on sensory inputs. Moreover, this temporal–spatial mapping advances our understanding of how attention and deviance detection processes unfold across time and space in the brain, refining existing models of auditory perception [78,79].

Only one previous study has simultaneously recorded EROS and ERPs to investigate DLPFC activity during a passive auditory oddball task [58]. The present study extends that work in several important ways. Technically, the sampling rate for optical signals was increased from 62.5 Hz to 125 Hz, and the number of EEG recording sites was expanded from 7 to 64, allowing for a more detailed topographical analysis of ERPs. Experimentally, participants’ attention was maintained on a fixation cross throughout the presentation of tones, and the probability of deviant stimulus presentation was reduced from 20% to 8%, all while maintaining the passive nature of the task. Overall, our findings suggest that the enhanced recording techniques reveal temporally co-occurring neurophysiological effects associated with auditory change detection. The results further support evidence indicating a role for left BA 46 in generating the MMN and for right BA 8 in generating the P3a ERP components [80,81,82,83,84,85,86,87].

### 4.1. Early Activation of the Left DLPFC (BA 46) and the Predictive Coding Framework

Our study’s observation of early activation in the left DLPFC, specifically BA 46, around 128 ms after the onset of deviant stimuli, highlights the DLPFC’s critical role in early auditory processing during a passive oddball task Low et al. [58]. Another study [57] recorded EROS only from the right hemisphere, focusing on the right superior frontal cortex and right inferior frontal cortex. Therefore, an activation in the left DLPFC could have been missed in that study. The early activation of BA 46 may reflect the pre-attentive integration of sensory inputs with the brain’s predictive model, indicating that even passive listening engages predictive mechanisms. This updating process is critical for adjusting future expectations and ensuring that the brain remains attuned to its environment, even in passive listening conditions, in order to minimize the discrepancy, or prediction error, between expected and actual stimuli.

The latency of the EROS peak closely matching the typical latency of MMN, a negative shift in frontocentral and central ERPs evoked by unexpected changes in tone frequency traditionally understood as a marker of pre-attentive auditory deviance detection [25,26,88,89,90], can be reinterpreted within the predictive coding framework [91,92,93,94]. Brodmann area 46 has long been associated with higher-order cognitive functions, including working memory and executive control [95,96,97,98,99] However, the early timing of BA 46 activation underscores the speed at which the predictive coding system operates. This finding align with the growing body of research indicating that the DLPFC is engaged in predictive modeling, even during a task that does not require conscious awareness of the deviant stimulus Predictive coding operates within a hierarchical framework of brain processing, where lower sensory areas generate bottom-up signals and higher cortical areas, like the DLPFC, generate top-down predictions. Our study’s findings suggest that BA 46, having reciprocal connections with the auditory cortex [6], is a crucial node in this hierarchy, where predictions are both generated and updated based on new sensory information. In the context of our oddball paradigm, the presentation of a deviant tone likely triggered bottom-up signals from the auditory cortex, which were then compared against the predictions generated by higher-order areas like BA 46. Future studies could investigate the pre-attentive activation in the left DLPFC as a function of participants’ handedness and as a function of more complex auditory signals, such as vowels, surrounding sounds or even words. The fact that BA 46 is activated during the MMN suggests that this region helps facilitate the transition from low-level prediction error detection to higher-order cognitive evaluations, which occur later during the P300 phase.

### 4.2. Late Activation of the Right DLPFC (BA 8) and Attention-Switching Theories

Our study observed significant late activation in the right DLPFC, particularly in BA 8, peaking approximately 312 ms after stimulus onset, corresponding with the P3a component of the ERP [27,29,30,88]. A similar pattern of activation associated with P300 was also reported by Low et al. [58]. However, in their study, the activation, which peaked at approximately 350 ms in a more anterior part of the DLPFC, was evoked only in the active version of the oddball task. Therefore, the slight differences in timing and activated ROI between our study and that by Low et al. [58] might reflect distinct roles of the right DLPFC in the task variants. In the context of the auditory oddball paradigm used in our study, the deviant tones represent unexpected events that violate the predicted sensory sequence. The activation of BA 8 during the P3a component likely reflects the brain’s detection of this deviation and the subsequent attentional processes, particularly the ability to reorient attention to infrequent deviant stimuli, particularly in passive listening tasks [4,27,100]. The involvement of BA 8 during these processes is supported by functional imaging [38,101], electrophysiological [102], and lesion studies [103], all of which highlight its critical role in the neural network underlying the P3a component. This activation is closely tied to attention-switching theories [104,105], which involve two primary cognitive mechanisms: the disengagement of attention from a previously attended stimulus and the reallocation of attention toward a new, more relevant stimulus. Attention-switching theories often differentiate between exogenous (bottom-up) and endogenous (top-down) mechanisms of attention control, as emphasized by models like the biased competition model [106,107] and the adaptive gain theory [108]. The late activation of BA 8 observed in our study aligns with exogenous attention switching, as participants were passively listening to auditory stimuli without the need for an active behavioral response. The deviant stimuli triggered an automatic reorientation of attention, reflected in the robust P3a component and BA 8 activation. Posner and Petersen’s [109] attention model divides attentional control into three networks: alerting, orienting, and executive control. The orienting network, in particular, is responsible for selecting specific information from sensory inputs, which is critical in the detection of novel or unexpected stimuli. Our findings align with this model, suggesting that BA 8 is a key node in the orienting network, rapidly shifting attention to deviant tones that require cognitive resources for further evaluation. By integrating these findings with existing models of attention, we provide further evidence of BA 8’s involvement in both automatic attentional reorientation and flexible cognitive processing, even in passive listening tasks.

### 4.3. Limitations

One key limitation of this study is the relatively small sample size, consisting of only 12 participants. While the use of detailed neuroimaging techniques, such as EROS and EEG, provides valuable insights into the neural dynamics underlying passive auditory deviance detection, the small number of subjects restricts the generalizability of the results. Small sample sizes increase the risk of sampling bias and reduce statistical power, making it difficult to confidently extrapolate the findings to a broader population. Additionally, the lack of diversity in participant characteristics, such as age and handedness, further limits the extent to which these results can be applied to other demographic groups. Future research with larger, more diverse samples is needed to validate these findings and ensure that the conclusions drawn are robust and widely applicable.

The electrical fields generated by the combined activity of thousands of neurons, particularly the postsynaptic potentials of cortical pyramidal cells [110], propagate through brain tissue, pass through the meninges and skull, and the attenuated signals that reach the scalp are detected by EEG electrodes placed on the surface of the scalp [65]. In this study, we increased the number of EEG electrode recording sites compared to previous studies that combined EROS and ERP recordings. This enhancement provides a significant advantage by offering more detailed spatial information for EEG analysis. However, it also imposes constraints on the placement of the optical setup, which had to be positioned between the electrode sites. EROS, measured by frequency-domain functional near-infrared spectroscopy (FD-fNIRS), capture fast optical signals based on the phase shift of near-infrared light pulses. As these light pulses pass through the brain tissue, they interact with neural structures, leading to a phase shift relative to the initial phase of the emitted light [45,111,112]. These phase delay measurements increase the sensitivity of EROS, making the technique highly effective for studying cortical surface activity, though it is less effective for investigating deeper brain structures or cortical sulci. Additionally, individual anatomical differences, such as skull thickness and density, can affect the depth of light penetration. Despite these limitations, FD-fNIRS offers higher temporal resolution than fMRI and better spatial resolution than EEG, making it well-suited for localizing neural activity in cortical gyri [113]. In some EROS studies, spatial resolution has been improved by conducting multiple sessions of optical data collection, each with different channel locations [48,54,57,58]. However, this approach can lead to habituation effects, which are difficult to control [30]. To avoid this issue, we performed the complete EROS montage in a single session per participant, with the maximum number of channels to 64. As a result, our EROS spatial resolution was less restricted compared to that of Low et al. [58]. These methodological improvements ensure robust data quality while addressing potential challenges inherent to combined EEG and EROS recordings.

## 5. Conclusions

In this study, the passive auditory oddball paradigm elicited ERP effects related to both the MMN and the P300 component. Specifically, early left DLPFC activation in BA 46 detected with EROS could be linked to pre-attentive mismatch detection (prediction error processing), while later activation in the right DLPFC (BA 8) might relate to the cognitive updating processes involved in predictive coding. The fact that BA 46 is activated during the MMN suggests that this region helps facilitate the transition from low-level prediction error detection to higher-order cognitive evaluations, which occur later during the P300 phase. The simultaneous observation of auditory deviance perception effects in both EROS and ERP measurements not only highlights the functional specificity of DLPFC regions in cognitive processing but also emphasizes the value of integrating EROS and ERP techniques as a powerful tool for mapping brain dynamics.

## Figures and Tables

**Table 1:** Comparison of raw and standardized data fNIRS contrasts, where Z> 2.575 and n > 10. (*) p< 0.05 after correction for multiple comparison.

Peak Latency (ms)	Talairach Coordinates (Peak Voxel)	Interval of Interest (ms)	Talairach Coordinates (Range of Interest)	Z-Score		$Z_{\mathrm{crit}}$	ROI
*Raw data*							
88	*x*: −13	72–104	*x*: [−18,−5]	2.95	<	3.11	BA 9
	*y*: 45		*y*: [ 43,50]				SFG
120	*x*: −50	112–136	*x*: [−53,−45]	2.76 *	>	2.67	BA 46
	*y*: 33		*y*: [ 33,35]				MFG
312	*x*: 32	296–320	*x*: [ 30,40]	2.91 *	>	2.81	BA 8
	*y*: 22		*y*: [ 20,28]				MFG, SFG
*Standardized data*							
128	*x*: −51	112–136	*x*: [−53,−40]	2.81 *	>	2.69	BA 46
	*y*: 32		*y*: [ 33,40]				MFG
312	*x*: 29	296–320	*x*: [ 28,38]	2.88 *	>	2.65	BA 8
	*y*: 21		*y*: [ 18,28]				MFG, SFG

## Data Availability

All data have been processed following the data security guidelines of the University of Lausanne for human participants. Privacy is regulated by the new Swiss Federal Act on Federal Data Protection (nFADP) (Status of 1 September 2023).
